# Bigger Is Better: Characteristics of Round Gobies Forming an Invasion Front in the Danube River

**DOI:** 10.1371/journal.pone.0073036

**Published:** 2013-09-09

**Authors:** Joerg Brandner, Alexander F. Cerwenka, Ulrich K. Schliewen, Juergen Geist

**Affiliations:** 1 Aquatic Systems Biology Unit, Department of Ecology and Ecosystem Management, Technische Universität München (TUM), Freising, Germany; 2 Department of Ichthyology, Bavarian State Collection of Zoology (ZSM), München, Germany; Aristotle University of Thessaloniki, Greece

## Abstract

Few studies have systematically investigated differences in performance, morphology and parasitic load of invaders at different stages of an invasion. This study analyzed phenotype-environment correlations in a fish invasion from initial absence until establishment in the headwater reach of the second largest European river, the Danube. Here, the round goby (*Neogobius melanostomus*) formed 73% of the fish abundance and 58% of the fish biomass in rip-rap bank habitats after establishment. The time from invasion until establishment was only about two years, indicating rapid expansion. Founder populations from the invasion front were different from longer established round goby populations in demography, morphology, feeding behaviour, sex ratio and parasitic load, indicating that plasticity in these traits determines invasion success. Competitive ability was mostly dependent on growth/size-related traits rather than on fecundity. As revealed by stable isotope analyses, specimens at the invasion front had a higher trophic position in the food web and seem to benefit from lower food competition. Somatic performance seems to be more important than investment in reproduction during the early stages of the invasion process and upstream-directed range expansion is not caused by out-migrating weak or juvenile individuals that were forced to leave high density areas due to high competition. This mechanism might be true for downstream introductions via drift. Greater abundance and densities of acanthocephalan endoparasites were observed at the invasion front, which contradicts the expectation that invasion success is determined by lower parasitic pressure in newly invaded areas. Overall, the pronounced changes in fish and invertebrate communities with a dominance of alien species suggest invasional meltdown and a shift of the upper Danube River towards a novel ecosystem with species that have greater resistance to goby predation. This seems to contribute to overcoming biological resistance and improve rapidity of dispersal.

## Introduction

Invasive species are important drivers of global biodiversity loss [1], [2] and one of the major threats to global freshwater biodiversity [3]–[5]. Successful invaders are not a random selection of species [6]. Instead, they often have certain life history traits in common, including a generalist feeding strategy, complex reproductive behavior involving e.g. nest guarding, the ability of rapid range expansion but also aspects of population structure, genetics and habitat use (e.g., [7]–[9]). Most of these successful invaders, including the zebra mussel *Dreissena polymorpha* Pallas, 1771 and the so-called ‘killer shrimp’ *Dikerogammarus villosus* (Sovinskij, 1894) have been blamed for serious ecosystem impacts worldwide [10]–[11]. Current studies identified plasticity in life history traits to be an important advantage to the success of invasive species, allowing them to easily adapt to different environments throughout the different stages of the invasion process [12]–[16]. Since several evolutionary and ecological processes can change life history strategies of invaders advancing from one stage to the next [17]–[19], time since invasion needs to be considered to identify and quantify the role of these factors [20]. However, to our knowledge, no study has yet systematically investigated biological invasion processes from total absence until the dominance of an invasive species, focusing on life history plasticity over time.

Recently, a benthic Ponto-Caspian gobiid fish species (Teleostei: Gobiidae), the round goby *Neogobius melanostomus* (Pallas, 1814), has colonized both freshwater and marine ecosystems on both sides of the Atlantic Ocean [21]. Its rapid spread and the high potential to cause ecological regime-shifts (e.g., [22]–[25]) have mobilized substantial scientific interest in this species as a model to study invasion biology processes worldwide (reviewed in Kornis et al., 2012 [26]). In the last two decades, an increasing number of rapid range expansions of *N. melanostomus* have been reported from the Laurentian Great Lakes watershed [21], [27]–[31], from almost the entire Baltic Sea region [32]–[34] and from many other large European waterbodies, including the Danube River [13], [35], [36] and the River Rhine [37]. Secondary invasions aside of the main navigation routes and migration corridors (e.g., [38]) and the proceeding spread of round goby worldwide highlight a new quality of potential threats especially to areas with high endemic aquatic biodiversity [39]. In lotic habitats, round goby was found to comprise more than 50% of the total fish catch [40], illustrating the potential impact on aquatic food webs. Therefore, a better knowledge of round goby ecology at all stages of the invasion is crucial to estimate associated ecosystem impacts [41].

Comparisons between native and non-native round goby populations revealed differences in distribution and abundance [42], as well as in the capability to generate phenotypic differences in life history traits. In particular, shifts in population characteristics, somatic condition, growth rate, diet and maturity as well as in external morphology were observed between the native range and newly invaded habitats [43]–[45]. However, none of these studies considered spatio-temporal effects on plasticity. Recent studies from the Trent-Severn-Waterway (Ontario, Canada) characterized initially invaded areas, subsequently referred to as “invasion front”, by having a lower proportion of sites containing round gobies, lower densities, larger individuals and male-biased sex-ratios [8], [46]. Consequently, demographics, life history-traits and growth at newly invaded areas seem to differ from relatively long-established areas. To date, little is known about invasions that are independent from ballast water transport and ship hull transfer. Additionally, population dynamics and life-history characteristics of round goby pioneers from other habitats such as large rivers are underrepresented in scientific studies on this topic.

In the German section of the Danube River, which is one of the most important European long-distance dispersal routes for aquatic invasive species [47], [48], *N. melanostomus* was first recorded in 2004 [49]. Here, round goby can be found both at established areas in densities of up to 20 individuals per square meter and not far upstream, at a distinct invasion front [13], where introduction is not directly related to navigational vessel traffic. Thus, the present invasion of *N. melanostomus* within the upper Danube River offered the opportunity to quantitatively study early (introduction) and late (establishment, spread and impact) phases of a round goby invasion. In particular, the greater availability of food resources and habitat structures in newly invaded areas may cause differences in demographic parameters of round goby populations such as length and weight distributions, and the proportion of sexes, but also in feeding behaviour, reproduction, parasitic load and fitness compared to areas with established populations. The great degree of phenotypic plasticity in *N. melanostomus* among distinct geographical regions is evident from several life trait variables such as length-weight relationships with b-values varying between 2.4 in the Sea of Azov [50] and 3.3 in the Sea of Marmara [51]. On the other hand, there are currently very few field studies available that were able to compare the plasticity of invasive species within the same ecosystem or habitat over time by comparing populations or sub-populations at recently invaded sites with established ones within the same system. This is, to our knowledge, the first study, examining a recent round goby invasion from total absence to the first occurrence until establishment.

The general objectives of this study were to (i) compare early and late phases of a round goby invasion at population- and specimen-level in a recently invaded, lotic ecosystem, (ii) test for phenotypic differences (length, weight and condition factor, hepato-somatic and gonado-somatic indices) between fish representing those early and late population stages, and (iii) analyze founder traits and demographic effects with respect to the time since invasion, considering abundance, sex ratio, parasitic load, and feeding patterns. Analogous to invasive plants [52] this study hypothesized that also animal invaders from recently invaded areas differ from their conspecifics in established populations by possessing an increased competitive ability, including greater body sizes and condition factors, reduced parasitic load and different feeding strategies. This study compared phenotypic characteristics of round gobies in pioneering and established populations within one of the most important European invasion pathways, the Danube River.

## Materials and Methods

### Ethics Statement

All specimens in the current study were sampled using electrofishing, which was conducted under license number 31-7563/2 to the Aquatic Systems Biology Unit of Technische Universität München (TUM). All specimens used for analyses were killed using an overdose of anaesthetic and immediately frozen on dry ice to avoid degradation of gut contents and muscle tissue. Following federal fishing laws and sampling licensing, all invasive gobies were removed from the Danube River, whereas all native fishes were carefully returned to the river after sampling. All efforts were carried out in strict accordance with the legal obligations of the Federal Republic of Germany.

### Field sampling

**Figure 1:**
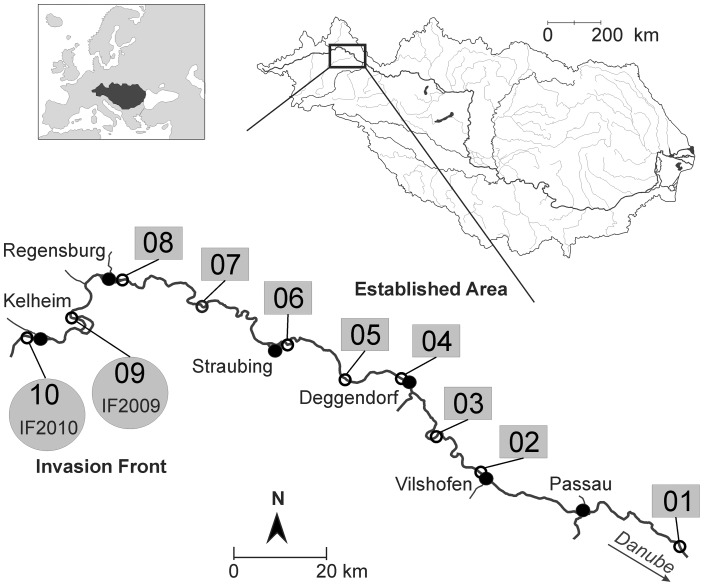
Study area at the upper Danube River in Austria and Germany. Study area with ten representatively chosen rip-rap sampling stretches covering a recent round goby invasion along the headwater reaches of the upper Danube River in Austria and Germany. The consecutive numbers denote two newly colonizing sub-populations at a recent invasion front (grey circles) “IF2010” (sampling stretch #10, first record: September 2010) and “IF2009” (sampling stretch #09, first record: August 2009) as well as eight established sub-populations from the “established area” (sampling stretches #08 to #01; grey rectangles). The Danube basin and the location of the study area within the drainage area are highlighted. Filled black circles denote important cities along the Danube River.

To explore potential differences between newly invaded and established “populations” (i.e. sub-populations *in sensu stricto)*, round goby distribution along a 200 river-km invasion pathway in the upper Danube River was monitored during a pilot study. In summer 2009, this investigation was conducted to identify the upstream border to which round gobies had reached (“invasion front”). Analogously to the sampling of Bronnenhuber et al. (2011) [30] at three Great Lakes tributaries, round gobies were considered absent at a site where no individuals were caught at a minimum of 1200 electroshocking seconds. The uppermost site where single individuals of *N. melanostomus* had been recorded (August 25^th^, 2009) was river-km 2,390.2 (48°58′39.03″N; 12°02′16.72″E). The intended sampling design comprised three river sections with an “established area”, where round goby had been recorded for the first time before 1^st^ January 2007, an “invasion front”, where a round goby invasion was expected to happen soon after the initiation of this study, and an uppermost “negative control area” with round goby absence during this study. Considering these findings, ten representatively distributed river stretches along the upper Danube River were selected (Figure 1, Table 1). The established area comprised eight river stretches (populations #01 to #08) from Engelhartszell (Austria) to the city of Regensburg (Germany). As round goby started to invade the river stretch #09 “Bad Abbach” in autumn 2009, this area was defined “IF2009” (invasion front 2009). Due to round goby invasion in the intended negative control area #10 “Kelheim” in autumn 2010, this river stretch was defined “IF2010” (invasion front 2010).

**Table 1:** Sampling design and location of river stretches.

Sampling Design				*Neogobius melanostomus*		Lower Boundary		Upper Boundary	
#	River Stretch	n	PAS	First Record	Population Status	rkm	GPS	rkm	GPS
10	Kelheim	10	300	2010[^1^]	newly colonizing	2,409	E 11°56′27′′	2,418	E 11°50′12′′
							N 48°54′29′′		N 48°54′01′′
09	Bad Abbach	10	301	2009[^1^]	newly colonizing	2,393	E 12°00′13′′	2,400	E 12°02′05′′
							N 48°57′57′′		N 48°56′03′′
08	Regensburg	9	270	u	established	2,373	E 12°10′41′′	2,377	E 12°08′29′′
							N 49°00′34′′		N 49°01′22′′
07	Geisling	6	180	u	established	2,350	E 12°23′37′′	2,354	E 12°21′02′′
							N 48°58′51′′		N 48°58′36′′
06	Straubing	4	110	2004[^2^]	established	2,309	E 12°42′26′′	2,317	E 12°36′56′′
							N 48°53′34′′		N 48°53′49′′
05	Mariaposching	5	131	u	established	2,292	E 12°52′12′′	2,298	E 12°47′46′′
							N 48°50′28′′		N 48°49′33′′
04	Deggendorf	3	90	u	established	2,280	E 12°59′50′′	2,289	E 12°54′26′′
							N 48°47′31′′		N 48°50′40′′
03	Aichet	8	240	u	established	2,267	E 13°03′08′′	2,273	E 13°02′15′′
							N 48°43′37′′		N 48°44′32′′
02	Vilshofen	9	273	2004[^2^]	established	2,250	E 13°10′44′′	2,259	E 13°05′41′′
							N 48°38′24′′		N 48°41′02′′
01	Engelhartszell	8	240	2003[^3^]	established	2,196	E 13°46′29"	2,202	E 13°43′21"
							N 48°28′32"		N 48°30′48"

Consecutive number, name, total number of rip-rap samplings (n), total number of point abundance samples (PAS), population status (defined after time since invasion), first record of *N. melanostomus*, river-kilometers (rkm) and GPS-coordinates of upper and lower boundaries (sorted in upstream to downstream order) of ten representatively distributed rip-rap river stretches from both river shorelines along the upper Danube River. [^1^] own observations [^2^] Paintner & Seifert (2006), [^3^] Zauner (pers. com.) , u  =  exact time point uncertain, but first record clearly before 2007.

The sampling was conducted from October 2009 to October 2011, covering the early (March–June) and late (August–October) annual growth period of fish as suggested from previous studies (e.g., [13]). In order to avoid the introduction of a systematic sampling bias (e.g. due to trends in water temperatures), even and uneven river stretches were sampled consecutively (first even and then uneven numbered river stretches). According to Sindilariu et al. (2006) [53] and our own observations, rip-rap structures are the preferred habitat of invasive round goby in the Danube River, representing about ⅔ of the available bank habitat in the study area. Thus, to exclude a possible bias due to different mesohabitat structures, only rip-rap habitats were sampled.

Fishes were collected during daylight from shorelines (in ∼60 cm water depth) by electrofishing (ELT62-IID; Grassl GmbH, Berchtesgaden, Germany) using the point abundance sampling (PAS) technique [54], [55] with a duration of 10 s and a distance of 10 meters between individual points. Every river stretch comprised at least 30 PAS-points at both shorelines. In total, 2,135 PAS-points were collected at 72 rip-rap samplings (Table 1).

All fishes were determined to species level, counted, measured (*L*_T_ to nearest mm) and weighted (*M*_T_ to nearest 0.2 g). Sex of *N. melanostomus* was determined by an examination of the morphology of the urogenital papilla [26]. Since sex determination is unreliable for juveniles with *L*_T_<5 cm, this size class was excluded from sex-specific analyses.

All fish species were inspected for infection rates with ectoparasitic plathyhelminths of the genus *Rossicotrema spp.* (black spot disease) and assigned into four categories (0 = no black spots; 1 = few, i.e. <5; 2 = medium, i.e. 5–100; 3 = high, i.e. >100).

In addition to the demographic sampling for characterizations on the population level, 365 round goby specimens were collected (targeting two females and two males from every single river stretch) for characterization of specimen level data. This sample subset was size-class selected (target 8–12 cm), as many morphometric indices assume isometry of body proportions in fish of varying size (e.g., [56]) and stable isotope signatures in *N. melanostomus* can be influenced by ontogenetic diet shifts [13]. The mean total length (*L*_T_) of all chosen specimens was 9.82 cm (SD = 1.15 cm) with a slope (b) of the length-weight-regression of about 3.0 (b = 3.045; R^2^ = 0.927; p<0.001), indicating isometric growth [56] for the chosen specimens. To test for site-, sex- and season-specific differences in length-weight relationships and to assess the possibility of pooling samples within the established area, slope comparison of length-weight regressions were computed and tested using ANCOVA. No statistically significant differences between slopes were identified, with p-values >0.05 in all cases. Due to the spatial shift of the invasion front between 2010 and 2011, specimens from those two years were analyzed separately.

As several recent studies described ontogenetic diet shifts in *N. melanostomus* [13], [24], [27], [57], one additional sample of 16 specimens (*L*_T_ of 4–14 cm) was collected at an established population (#08, “Regensburg”; 49°01′01.95″ N; 12°09′21.09″ E) on October 15^th^, 2010, and another additional sample of 15 specimens (*L*_T_ of 8–17 cm) was collected at IF2010 (#10, “Kelheim”; 48°54′26.99″N; 11°53′24.56″E) on September 9^th^, 2011 (Figure 1, Table 1) to test for this size effect at different stages of the invasion process. All specimens were deposited at the ichthyological collection of the Bavarian State Collection of Zoology (ZSM).

The wet weights of liver, gut contents, ovaries in females, testes and seminal vesicles in males were recorded to the nearest 0.001 g. As round goby is known to serve as a paratenic host for acanthocephalans [58], [59], subadult acanthocephalans attached to liver, kidney, spleen, gonads and the surface of the intestinal tract were counted using a stereo-microscope. In order to test the “enemy release-hypothesis”, suggesting that invasive species carry less parasites in newly invaded areas than in established or original areas of distribution (e.g., [60]), ecological indicators of parasite infection were applied according to Ondračková et al. (2005) [61], using mean abundance (i.e. mean number of parasites per host) and mean density (i.e. abundance per fish total mass).

### Stable isotope analysis

To obtain markers for middle to long-term feeding patterns, δ^13^C and δ^15^N stable isotope analyses of round goby flank muscle tissue (about 0.5–1.0 cm^3^, defatted with chloroform-methanol (2:1) solution) were conducted as described in Brandner et al. (2013) [13]. The additional sets of samples with greater length variation were analyzed to test for (i) correlations between *L*_T_ and δ^15^N signatures, and (ii) a diet shift between muscle tissue and gut contents. The δ^15^N values of the gut contents were calculated as averages weighted by their index of food importance (see *I*_FI_ below) from mean δ^15^N signatures of benthic invertebrates collected from the upper Danube River [13]. Repeated analyses of a solid internal laboratory standard (bovine horn, run after each ten samples) showed maximum standard deviations of 0.15 ‰ for δ^15^N and 0.15 ‰ for δ^13^C values.

### Fish gut analyses

Digestive tract dissection and processing was conducted following Brandner et al. (2013) [13] with the anterior digestive tract being weighted to the nearest 0.001 g before and after emptying to obtain the wet weight of gut contents. All food items from the digestive tract samples were fixed in ethanol, identified to the lowest possible taxon considering manageable taxonomic levels, counted and visually estimated to the nearest % proportion of volume, using a stereo microscope.

### Benthic invertebrates

Quantitative samples of benthic invertebrates were collected using a suction sampler (as described in Brandner et al., 2013 [13]) from the same sites where gobies were sampled (∼60 cm water depth, duration  = 120 s, three replicates). Altogether 250 samples of benthic invertebrates were preserved in 70% ethanol immediately after capture. A total of about 46,500 benthic invertebrates were identified to the lowest possible taxon considering manageable taxonomical levels. Organisms belonging to the same taxon or cumulative category were counted and expressed as catch per unit effort (CPUE [min^−1^]). The percent volumetric proportion of each taxon within a sample was visually estimated analogously to the gut analysis in fish.

### Indexing and statistical analyses

The somatic mass (*M*_S_) was calculated as *M*_S_  =  *M*_T_ – (*M*_indexed organ_ + *M*_g_) with *M*_g_  =  gut content mass to compute the following indices: To test for differences in important body mass indices between specimens of a population, the hepato-somatic index (HSI = 100 *M*_ liver_ *M*_S_^−1^) and the gonado-somatic index (GSI = 100 *M*_gonads_ *M*_S_^−1^) as a proxy of energetic investment into reproduction were calculated for both sexes [62]. Fulton's condition factor *K* was calculated as *K* = 100 (*M*_T_ – *M*_g_) *L*_T_^−3^ to assess length-weight relationships between populations and specimens [56]. To assess food uptake and to test for potential food limitation effects on feeding behaviour, the index of stomach fullness (*I*_SF_) was calculated following Hyslop (1980) [63] as *I*_SF_ = 100 *M*_g_ *M*_T_^−1^.

Analogously to Brandner et al. (2013) [13] the relative importance of a food item i among all items j (“index of food importance”) for a population was calculated as *I_FI_*_(_i_)_  = 100 *O*_(_i_)_ *V*_(_i_)_ (∑ *^j^_n = 1_ O*_(_i_)_ *V*_(_i_)_ ) ^−^1 with *O*  =  % occurance of prey i and *V*  =  % volume of prey i. *I*_FI_ varies from 0 to 100, with higher values corresponding to a larger contribution of one food item as compared to total gut content. Since benthic invertebrate samples were treated like gut content samples, importance of naturally available prey was also calculated following the above mentioned formula as “index of environmental importance” (*I*_EI_) for each food item i.

Dissimilarity-distances (squared Euclidian distance) between the 72 samplings from 10 river stretches were calculated using *L*_T_, *M*_T_ and *K* of females, males and juveniles, the proportions of females (as a relative sex ratio) and catch data (mean CPUE and frequency of occurrence (ƒ_O_) of *N. melanostomus*, the most abundant autochthonous fish species *Barbus barbus* (L., 1758) and *Squalius cephalus* (L., 1758) pooled as an indicator for abundant potential prey, and other fish species) from the corresponding rip-rap sampling sites as variables. The results were plotted as a two-dimensional non-metric multi-dimensional scaling (NMDS). In order to assess the importance of catch data, *L*_T_ and *M*_T_ as well as sex-ratio, additional NMDS analyses considering these factors separately were carried out. As water temperature, discharge and seasonal effects within the sampling procedure may influence all these parameters, additionally paired Kruskal-Wallis tests were conducted to analyze potential trends and differences between the established populations. Only occasional and unsystematic differences without any trends in single parameters among single established populations were detected. Since no significant differences between the slopes of the established populations were found, these data were pooled by the time since invasion (year of first record, Table 2).

**Table 2:** Population dynamics in *N. melanostomus* and bycatch at three areas (stages) of the invasion.

Sampling			Round Goby			Barbel & Chub		Other Fish Species	
Sampling Area	Season	PAS [n]	First Record	CPUE [PAS^−1^]	ƒ_O_ [%]	CPUE [PAS^−1^]	ƒ_O_ [%]	CPUE [PAS^−1^]	ƒ_O_ [%]
IF2010	late 2009	60	September 2010	nd	nd	1.73	63.3	0.40	30.0
	early 2010	60		nd	nd	2.47	76.7	0.35	31.7
	**late 2010**	**60**		**0.08**	**8.3**	**1.28**	**68.3**	**0.90**	**53.3**
	early 2011	60		0.08	5.0	1.50	56.7	0.63	43.3
	late 2011	60		1.63	63.3	2.05	78.3	2.00	63.3
IF2009	**late 2009**	**61**	August 2009	**0.08**	**6.6**	**0.15**	**9.8**	**1.69**	**44.1**
	early 2010	60		0.03	3.3	0.20	16.7	0.57	35.0
	late 2010	60		2.18	78.3	0.23	20.0	3.43	83.3
	early 2011	60		4.60	96.7	0.20	8.3	0.23	16.7
	late 2011	60		3.47	86.7	0.13	10.0	0.38	31.7
Established Area	late 2009	59	before 2007	4.61	89.2	0.04	4.3	0.70	40.7
	early 2010	425		2.05	73.1	0.06	4.3	0.34	23.9
	late 2010	306		3.68	75.8	0.30	14.7	1.46	37.9
	early 2011	444		4.52	89.0	0.08	3.7	0.52	32.8
	late 2011	300		5.50	92.0	0.17	11.3	0.72	41.3

The sampled rip-rap river stretches (upper Danube River, autumn 2009 to autumn 2011) were assigned to the three sampling areas “IF2010”, “IF2009”, “established area” using the time since invasion (year of first record), with the number of point abundance samples (PAS) and catch data of invasive round goby, barbel *Barbus barbus* & chub *Squalius cephalus* (pooled) as most abundant autochthonous fish species and other fish species (rest). The catch (using electrofishing with continuous DC, duration 10s per PAS) is explained as the mean catch per unit effort (CPUE) [PAS^−1^] and the mean frequency of occurrence (ƒ_O_) [%]. The abbreviation “nd” denotes “not detectable”. Data from the first time of occurrence are shown in bold.

Specimens of the established area (n = 298) had a mean *L*_T_ of 9.82 cm (SD = 1.09 cm) with a slope of the length-weight-regression of b = 3.02 (R^2^ = 0.91; p<0.001). Specimens of the IF2009 (n = 36) had a mean *L*_T_ of 9.35 cm (SD = 1.25 cm) with a slope of the length-weight-regression of b = 3.09 (R^2^ = 0.96; p<0.001). Specimens of the IF2010 (n = 31) had a mean *L*_T_ of 10.12 cm (SD = 1.41 cm) with a slope of the length-weight-regression of b = 3.11 (R^2^ = 0.98; p<0.001). ANCOVA comparisons of the slopes revealed no significant differences between these three groups (all p>0.05). As *L*_T_, *M*_T_, *K, I*_FI_, *I*_EI_, *I*_SF_, were not normally distributed (Shapiro-Wilk test), multiple comparisons between populations and specimens were computed using non-parametric Kruskal-Wallis tests followed by (post hoc) Mann-Whitney U pairwise tests (Bonferroni corrected). Mann-Whitney U tests were applied to analyze these metrics for potential sex-specific differences. Differences from an expected equilibrium in the distribution of males and females as well as potential differences in the distribution of males and females (sex ratio) between the sampling areas were tested using the chi-square test. Significance was accepted at p≤0.05 for all statistical tests. Statistical analyses and plots were computed using Statistica (version 6.1, StatSoft Inc., Tulsa, OK, USA).

## Results

### Fish community

A total of 30 fish species, comprising 9,138 specimens (mean CPUE  = 4.3 fish PAS^−1^) were recorded at 2,135 rip-rap PAS-points in the upper River Danube between autumn 2009 and autumn 2011. Round goby contributed 73% (n = 6,627) and a biomass of about 58% (62 kg) to the total catch. Round goby was found throughout the sampling area, except for the most upstream sampling stretch, where first invaders (four females and one male) were recorded in autumn 2010. With a proportion of 53.1% females to the total catch (n_♀_ = 3,205; n_♂_ = 2,835), the overall sex ratio (f : m = 1 : 0.88) was significantly (χ2, p<0.001) different from an expected equilibrium.

Other invasive Ponto-Caspian gobies like the bighead goby *Ponticola kessleri* (Günther, 1861) and the tubenose goby *Proterorhinus marmoratus* (Pallas, 1814) were found continuously but in much lower abundances (<3% of total catch). One specimen of the racer goby *Babka gymnotrachelus* (Kessler, 1857) was found at river stretch #05 “Mariaposching” as a first record in Germany [64]. Barbel *B. barbus* and chub *S. cephalus* were the most abundant autochthonous fish species detected in each of the three investigated areas in the upper Danube River comprising about 9% of the total catch. Other fish species mainly comprised ide *Leuciscus idus* (L., 1758), bleak *Alburnus alburnus* (L., 1758), common nase *Chondrostoma nasus* (L., 1758), European perch *Perca fluviatilis* (L., 1758) and pike-perch *Sander lucioperca* (L., 1758) as well as European eel *Anguilla anguilla* (L., 1758) and to some extent burbot *Lota lota* (L., 1758), Wels catfish *Silurus glanis* L., 1758 and northern pike *Esox lucius* L., 1758.

Species of high conservation priority such as zingel *Zingel zingel* (L., 1766) and the gudgeon species *Romanogobio vladikovy* (Fang, 1943) endemic to the Danube basin, as well as bullhead *Cottus gobio* L., 1758 and schneider *Alburnoides bipunctatus* (Bloch, 1782) were present in very low abundances and limited to the river stretches #06 and #01.

### Round goby population data

The CPUE of *N. melanostomus* differed significantly (Kruskal-Wallis, p<0.001) between investigated areas (Table 2). In the IF2010 population, the mean CPUE (mean  = 0.4 [PAS^−1^]; SE = 0.3 [PAS^−1^]) was about 10-fold (Mann-Whitney U, p<0.001) lower compared to the area where the species has been established for at least 30 months (mean = 3.9 [PAS^−1^]; SE = 0.4 [PAS^−1^]). No significant differences in the mean CPUE were observed neither between IF2009 (mean = 2.1 [PAS^−1^]; SD = 0.6 [PAS^−1^]) and IF2010, nor between IF2009 and the established area. The proportion of point abundance samples containing *N. melanostomus* (ƒ_O_) significantly (Kruskal-Wallis, p<0.001) differed between the investigated areas. In the established area, ƒ_O_ was significantly (Mann-Whitney U; p<0.001) higher (ƒ_O_ = 82%; SE = 3%) compared with the IF2010 (ƒ_O_ = 15%; SE = 10%). No difference in ƒ_O_ was observed between the established area and the IF2009 (ƒ_O_ = 54%; SE = 14%). In the established area, peak-abundances of 25 round goby PAS^−1^ (river stretch “07_Regensburg”; n = 1,490; mean = 5.9 PAS^−1^; SD = 3.6; ƒ_O_ = 97.4%) and 26 round goby PAS^−1^ (river stretch “02_Vilshofen”; n = 1,943; mean = 8.0 PAS^−1^; SD = 5.3; ƒ_O_ = 95.9%) were observed in autumn 2011.

At the IF2010, round goby CPUE (20-fold) and ƒ_O_ (8-fold) increased from the late season 2010 to 2011. Analogously, at the IF2009, CPUE (27-fold) and ƒ_O_ (12-fold) increased from the late season 2009 to 2010. In both cases, one year later (i.e. the second year after the first record), round goby population density had doubled, reaching values similar to those from the established area (Table 2).

The mean CPUE of barbel and chub significantly (Kruskal-Wallis, p<0.001) differed between the investigated areas being inversely related to goby abundance (Table 2). At the IF2010, the mean CPUE (mean = 1.8 [PAS^−1^]; SE = 0.2 [PAS^−1^]) of barbel and chub was significantly (Mann-Whitney U, p<0.001) higher (about 20-fold) as compared with the established area (mean = 0.1 [PAS^−1^]; SE = 0.03 [PAS^−1^]) and (about 10-fold) the IF2009 (mean = 0.2 [PAS^−1^]; SE = 0.02 [PAS^−1^]). No significant difference in the mean CPUE was observed between the IF2009 and the established area. Also, the mean ƒ_O_ of barbel and chub significantly (Kruskal-Wallis, p<0.001) differed between the investigated areas. At the IF2010, the mean ƒ_O_ was significantly (Mann-Whitney U; p<0.001) higher (ƒ_O_ = 69%; SE = 5%) as compared with the IF2009 (ƒ_O_ = 13%; SE = 2%) and the established area. At the IF2009, mean ƒ_O_ was significantly (Mann-Whitney U; p<0.05) higher (ƒ_O_ = 7%; SE = 1%) as compared with the established area.

**Figure 2:**
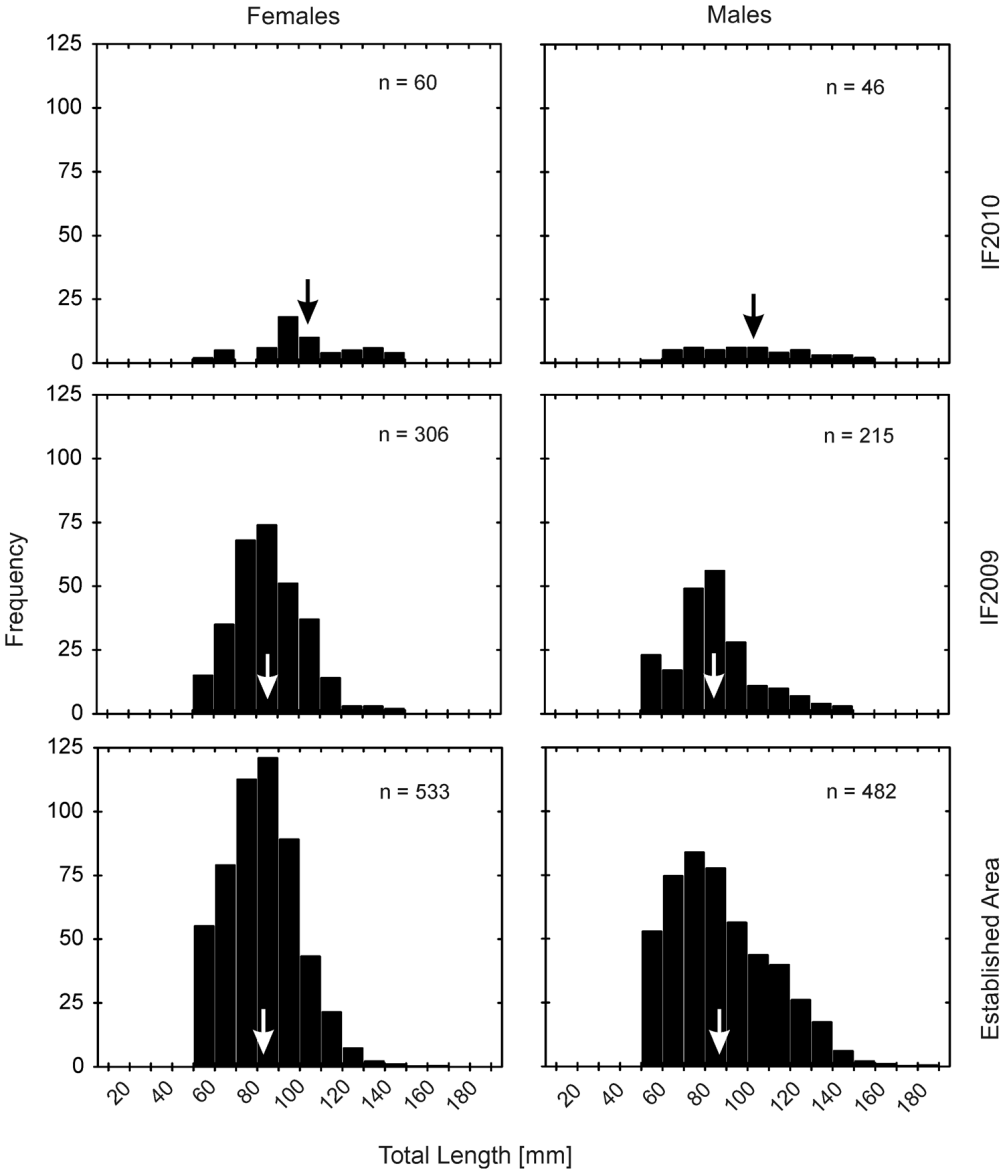
Length-frequency distributions of newly colonizing and established *N. melanostomus* populations. Length-frequency distributions (*L*_T_ in mm) of newly colonizing (invasion front 2010, “IF2010” and invasion front 2009, “IF2009”) and established (first record before 2007) round goby populations in the upper Danube River, Germany (left row: females; right row: males). The mean total lengths [mm] of each sex are indicated by arrows. The established area was normalised to 300 PAS-points to display identical catch effort in all populations. Note that juveniles (*L*_T_<5 cm) were excluded from sex-specific comparison since definite assignment of sex remained doubtful in many cases for this size class without dissection.

*L*_T_ of recorded *N. melanostomus* varied from 34 to 163 mm in females, and from 40 to 187 mm in males. The contribution of different length cohorts (Figure 2) was not normally distributed (Lilliefors, p*<*0.05) in each of the investigated populations. The largest individuals of each sex were captured in the established area as might be expected from the larger sample size (n = 5380; 99^th^ percentile of *L*_T_ = 14.2 cm). However, mean *L*_T_ was highest (Kruskal-Wallis, p<0.001) in the IF2010 (n = 108; 99^th^ percentile of *L*_T_ = 15.2 cm) in both sexes (Table 3, Figure 2). Females and males from the IF2010 were both significantly (Mann Whitney U, p<0.001) larger (both by about 20%) than in the IF2009 and the established area (Mann-Whitney U, p<0.001), by about 25% and 16%, respectively. Also, females from the IF2009 were significantly (Mann-Whitney U, p<0.01) larger than in the established area (Table 3). Males from the IF2009 were not significantly larger than those from the established area. Females were larger than their male conspecifics at the IF2010 and at the IF2009, but not significantly. Males however, were significantly (Mann-Whitney U, p<0.001) larger than females in the established area.

**Table 3:** Comparison of performance indicators of *N. melanostomus* at population level.

Population-Level			IF2010			IF2009			Established Area		
Performance Indicators		p	n	mean	SD	n	mean	SD	n	mean	SD
*L*_T_	females[cm]	***	60	10.4 ^a^	2.3	306	8.6 ^b^	1.7	2757	**8.3** ^c^	1.8
	males [cm]	***	46	10.2 ^a^	2.6	215	8.5 ^b^	2.0	2478	**8.8** ^b^	2.4
	juveniles [cm]	ns	2	4.6 ^a^	0.1	95	4.3 ^a^	0.4	633	4.3^ a^	0.5
*M*_T_	females[g]	***	60	20.2 ^a^	13.0	306	10.4 ^b^	7.3	2757	**9.0** ^c^	6.5
	males [g]	***	46	18.5 ^a^	14.4	215	10.3 ^b^	8.6	2478	**11.7** ^b^	10.6
	juveniles [g]	ns	2	1.4 ^a^	0.3	95	1.0 ^a^	0.3	633	1.0 ^a^	0.4
*K*	females	***	60	**1.56** ^ a^	0.14	306	**1.44** ^ b^	0.18	2757	**1.38** ^c^	0.18
	males	***	46	**1.50** ^a^	0.12	215	**1.41** ^b^	0.16	2478	**1.36** ^c^	0.17
	juveniles	ns	2	1.40 ^a^	0.16	95	1.22 ^a^	0.24	633	1.23 ^a^	0.27
Proportion of	females [%]		60	56.6		**306**	58.7		**2757**	52.7	
	males [%]		46	43.4		**215**	41.3		**2478**	47.3	
Overall sex ratio	f : m	***	106	1:0.77		571	*1:0.70*		5235	*1:0.90*	

72 sub-populations from the upper Danube River were assigned to the categories “IF2010” (invasion front 2010, 10 sub-populations), “IF2009” (invasion front 2009, 10 sub-populations) and “established area” (52 sub-populations) using time since invasion (see Table 2). Numbers of fish analyzed, means and corresponding standard deviations (SD) of total length (*L*_T_), total body mass (*M*_T_) and Fulton's condition factor (*K*) are displayed for both sexes and for juveniles (*L*_T_<5 cm). Relative proportions of females and males, as well as the overall sex-ratio were calculated from the total catch (excluding juveniles) of the sub-populations, respectively. Superscript letters denote significant differences (Kruskal-Wallis test) with p-values encoded by asterisks (*denotes p≤0.05; ** denotes p<0.01; *** denotes p<0.001). Values highlighted in bold denote significant (Mann-Whitney U test) differences between sexes. Values in italics denote significant (χ^2^ test) differences in the contribution of sexes between sampling areas.

*M*_T_ of *N. melanostomus* varied from 0.4 to 63.0 g in females, and from 0.4 to 98.4 g in males. Analogously to the trends observed in *L*_T_, the heaviest individuals of each sex were captured in the established area. However, both in females and males, the mean *M*_T_ was highest (Kruskal-Wallis, p<0.001) in the IF2010 population. In females, *M*_T_ from the IF2010 was both significantly (Mann-Whitney U, p<0.001) higher than in the IF2009 and the established populations. *M*_T_ of females from the IF2009 was significantly (Mann-Whitney U, p<0.01) higher than in the established area (Table 3). Also in males, *M*_T_ from the IF2010 was significantly (Mann-Whitney U, p<0.001) higher than in both IF2009 and established area. Males from the IF2009 were not significantly heavier than those from the established area (Table 3). Females were heavier than their male conspecifics at the IF2010 and at the IF2009, however not significantly. Males however, were significantly (Mann-Whitney U, p<0.001) heavier than females in the established area.

Both in females and males, *K* significantly (Kruskal-Wallis, p<0.001) differed between the investigated populations. In females, the highest value (Mann-Whitney U, p<0.001) was recorded at the IF2010, a medium value (Mann-Whitney U, p<0.001) at the IF2009, and the lowest value (Mann-Whitney U, p<0.001) in the established area. Also in males, *K* was highest (Mann-Whitney U, p<0.001) at the IF2010, medium (Mann-Whitney U, p<0.001) at the IF2009 and lowest (Mann-Whitney U, p<0.01) at the established area (Table 3). In females, *K* was significantly higher than in males at the IF2010 (Mann-Whitney U, p<0.05), at the IF2009 (Mann-Whitney U, p<0.05) and at the established area (Mann-Whitney U, p<0.001).

The proportion of fish smaller than 5 cm (juveniles) was 2% in the IF2010, 16% in the IF2009 and 9% in the established area. No juveniles in this size-class were recorded at IF2010 and at IF2009 when round goby had been detected there for the first time.

All areas investigated were female-dominated, with differences from equilibrium being significant at the IF2009 (χ^2^, p<0.001) and the established area (χ^2^, p<0.001) (Table 3). Both at the IF2010 and the IF2009, relatively higher proportions of females were observed than in the established area. However, this female-dominated pattern in the distribution of sexes was only significantly different (χ^2^; p<0.01) between IF2009 and the established area (Table 3).

**Figure 3:**
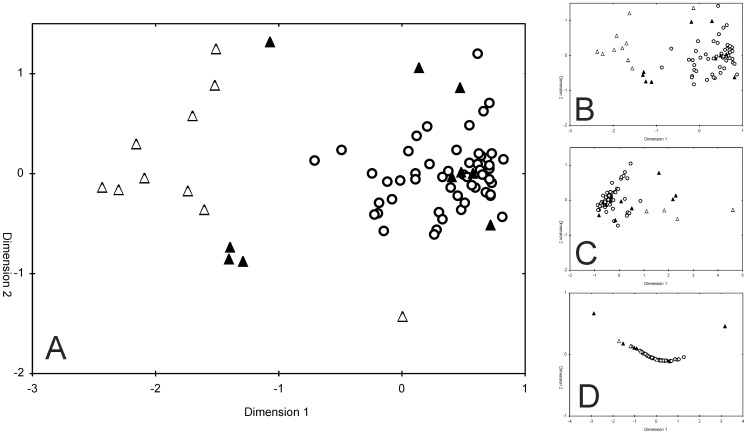
Nonmetric multidimensional scaling of *N. melanostomus* performance metrics. Nonmetric multidimensional scaling (NMDS) of *N. melanostomus* population-specific performance metrics calculated from point-abundance sampling data (autumn 2009 – autumn 2011). Dissimilarity-distances between 72 samplings from 10 river stretches (rip-rap habitats) were calculated using the squared Euclidian distance and displayed by triangles (invasion front 2010, “IF2010”), filled triangles (invasion front 2009, “IF2009”) and circles (established populations). *L*_T_(f), *L*_T_(m), *L*_T_(j), *M*_T_(f), *M*_T_(m), *M*_T_(j), *K*(f), *K*(m), *K*(j), proportion of females and catch data (mean CPUE and frequency of occurrence of (i) *N. melanostomus*, (ii) *Barbus barbus* and *Squalius cephalus* (combined) and (iii) other fish species from the corresponding sampling sites were used as variables in panel A (stress  = 0.10). Catch data were analyzed in panel B (stress = 0.11). *L*_T_(f), *L*_T_(m), *L*_T_(j), *M*_T_(f), *M*_T_(m), *M*_T_(j), were analyzed in panel C (stress = 0.08) and the sex ratio (proportions of females and males) was analyzed in panel D (stress = 0.001).

An analysis of the 72 *N. melanostomus* samplings using NMDS revealed two clusters, with a separation of invasion front samples from established ones (Figure 3A), particularly for the 2010 data. The main factors underlying this pattern were found to be catch data, length and weight differences, whereas the sex ratio was not important (Figure 3B, C, D). This pattern remained stable, independent of using arithmetic mean or median values (data not shown) as input variables in the analyses. Overall, differences between established and invasion front populations were far more pronounced than differences among all established populations from different locations.

### Round goby specimen data

In females, the mean GSI differed between the investigated areas (Kruskal-Wallis; p<0.001) with lowest values at the IF2010. At the IF2010, female GSI was significantly lower than in the IF2009 (Mann-Whitney U; p<0.001) and the established populations (Mann-Whitney U; p<0.05). Also in males, the GSI was lowest in the IF2010, however differences were not significant (Table 4).

**Table 4:** Comparison of performance indicators of *N. melanostomus* at specimen level.

Specimen-Level			IF2010			IF2009			Established Area		
Performance Indicators		p	n	mean	SD	n	mean	SD	n	mean	SD
Fecundity and condition	GSI_ females_	***	18	2.0^ a^	3.3	18	3.6^ b^	4.1	145	4.3^ b^	5.0
	GSI _males_	ns	13	0.1	0.1	18	0.7	1.7	153	0.3	0.8
	*K*_ females_	**	18	1.51 **^a^**	0.09	17	1.47 ^b^	0.15	140	1.41^b^	0.14
	*K*_males_	*	12	1.47 **^a^**	0.07	17	1.38^ b^	0.09	145	1.39^ b^	0.12
	HSI_ females_	***	18	**6.5^ a^**	1.3	6	3.2^ b^	0.90	76	4.6^ c^	1.3
	HSI_ males_	**	12	**5.4^ a^**	0.7	6	3.4^ b^	0.8	74	4.4^ b^	1.3
Stable isotopes	δ^15^N _females_ [‰]	***	17	15.59^ a^	0.40	18	15.89^ a^	0.50	146	**14.91** ^ b^	0.60
	δ^15^N _males_ [‰]	***	13	15.38^ a^	0.35	18	15.93^ b^	0.54	152	**14.75** ^ c^	0.57
	δ^13^C_ females_ [‰]	ns	17	−29.34	−0.42	18	**−29.11**	−0.55	146	−28.96	−0.86
	δ^13^C_ males_ [‰]	ns	13	−29.37	−0.52	18	**−29.45**	−0.40	152	−29.08	−0.87
Feeding and prey-specific indices	*I*_SF_	ns	31	2.8	0.8	34	3.4	1.6	285	3.1	1.4
	*I*_FI (EPT)_	**	33	7.7 **^a^**	22.9	46	5.3 ^b^	19.5	500	0.6 ^b^	5.1
	CPUE _(EPT)_ [min^−1^]	***	24	1.4^ a^	1.22	30	0.3^ b^	0.45	161	0.2^ b^	0.56
	*I*_EI (EPT)_	***	24	3.1^ a^	8.5	30	1.1^ b^	2.9	161	0.1^ c^	0.1
Endoparasites (Acanthocephala)	abundance [n] females	***	18	**108** ^ a^	54	18	20^ b^	17	146	49 ^b^	68
	abundance [n] males	***	13	**57** ^ a^	27	18	10^ b^	15	152	36^ c^	53
	density [n/g] females	***	18	6.4^ a^	3.5	18	**1.4** ^ b^	1.2	146	**3.3** ^ b^	4.0
	density [n/g] males	***	13	4.3^ a^	2.1	18	**0.7** ^ b^	0.7	152	**2.4** ^ c^	2.9
Ectoparasites (*Rossicotrema* spp.)	abundance [0–3] females	ns	18	0.06	0.24	18	0.00	0.00	146	0.08	0.29
	abundance [0–3] males	ns	13	0.15	0.38	18	0.00	0.00	152	0.07	0.30

365 specimens (mean *L*_T_ = 9.8 cm; SD = 1.2 cm) originating from the investigated sub-populations “IF2010” (invasion front 2010), “IF2009” (invasion front 2009) and “established area” along the upper Danube River (early season 2010 – late season 2011) were sampled for analyses. Numbers of fish dissected, means and corresponding standard deviations (SD) of fecundity and condition indices (gonado-somatic index GSI, Fulton's Condition Factor *K*, hepato-somatic index HSI), stable isotope signatures (δ^15^N, δ^13^C), feeding indices (index of stomach fullness *I*_SF_; index of food importance of ephemeroptera, trichoptera and plecoptera *I*_FI (EPT)_) and prey-specific indices (catch per unit effort CPUE _(EPT)_ and index of environmental importance of ephemeroptera, trichoptera and plecoptera *I*_EI (EPT)_) and parasite infection indices were calculated for females and males. Values highlighted in bold denote significant differences (Mann-Whitney U test) between sexes. Superscript letters denote significant differences (Kruskal-Wallis test) between populations with p-values encoded by asterisks (*denotes p≤0.05; ** denotes p<0.01; *** denotes p<0.001).

Fulton's Condition factor significantly differed between the investigated populations both in females (Kruskal-Wallis; p<0.01) and males (Kruskal-Wallis; p<0.05) with highest values at the invasion front. In females, *K* was significantly (Mann-Whitney U; p<0.01) higher at the IF2010 compared with the established area. Also in males, *K* was both significantly (Mann-Whitney U; p<0.05) higher at the IF2010 compared with the IF2009 and the established area. No significant sex-specific differences in *K* were observed within the populations analyzed.

The mean δ^15^N values in both females and males significantly (Kruskal-Wallis; p<0.001) differed between the investigated areas, with lowest values in the established area and highest values at the IF2009. Compared with the established area, in females, mean δ^15^N values were significantly (Mann-Whitney U; p<0.001) higher both at the IF2010 (Δ _δ15N_ = 0.7 [‰]) and the IF2009 (Δ _δ15N_ = 1.0 [‰]). No significant differences in female δ^15^N values were observed between the IF2010 and the IF2009. Also in males, the mean δ^15^N-values were significantly (Mann-Whitney U; p<0.001) higher at the IF2010 (Δ _δ15N_ = 0.6 [‰]) and the IF2009 (Δ _δ15N_ = 1.2 [‰]), compared with the established area. In case of males, mean δ^15^N values significantly (Mann-Whitney U; p<0.001) differed between the IF2010 and the IF2009. A significant (Mann-Whitney U; p<0.01) sex-specific difference between females and males in the mean δ^15^N value was observed in the established area only, where females had lower δ^15^N values than males.

**Figure 4:**
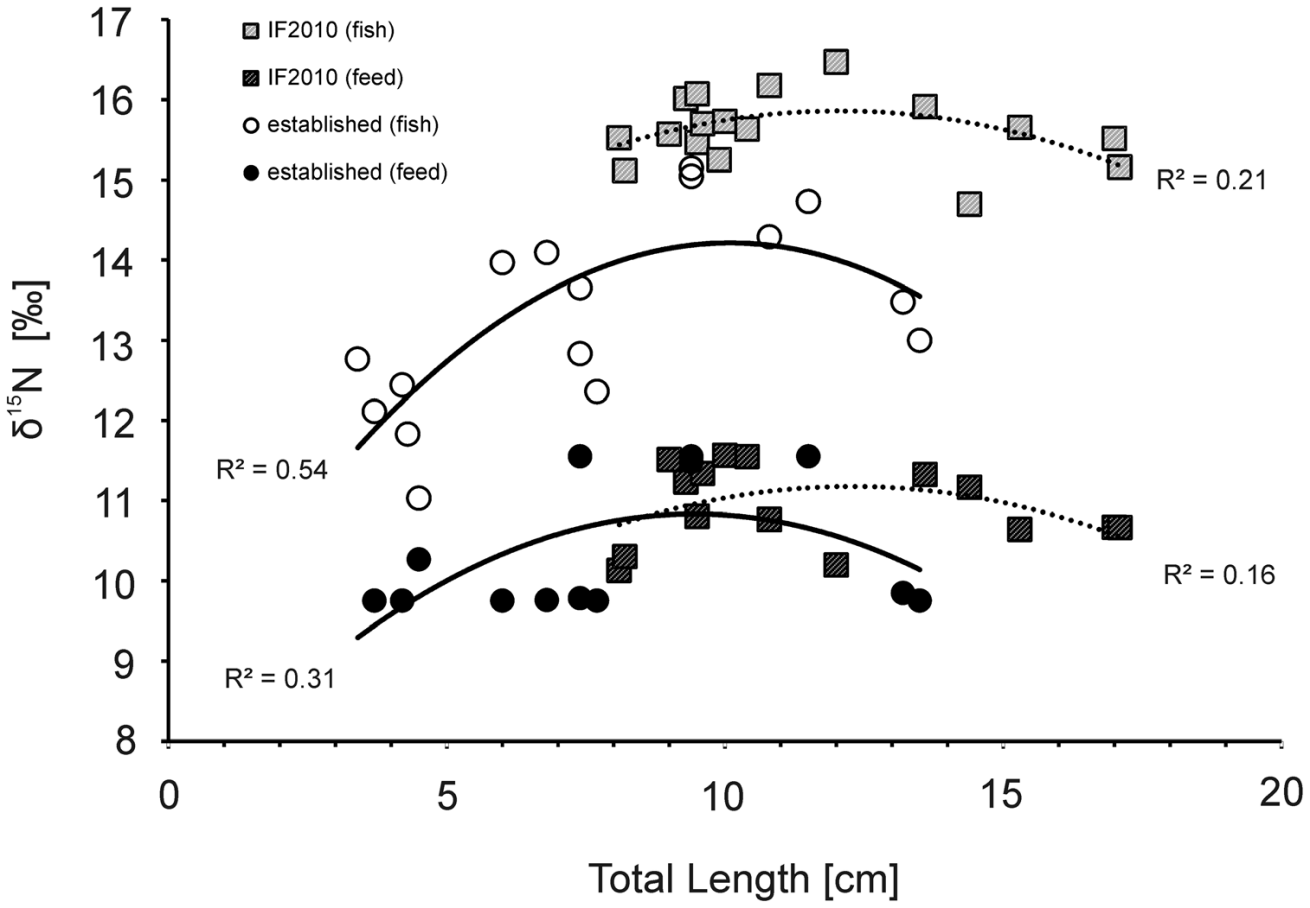
Diet-tissue shift and ontogenetic dietary shift in *N. melanostomus*. Changes (diet-tissue shift, ontogenetic dietary shift) in the relative nitrogen isotope ratio of gut contents (“Feed”, filled symbols) and muscle tissue of *N. melanostomus* (“Fish”, open symbols) from the invasion front 2010 (“IF2010”, squares, dashed lines) and the “established area” (circles, continuous lines) are displayed in relation to the total length. Lines are parabolic regressions (p<0.001) based on total length and the type of tissue (with R^2^ given in the diagram).

The δ^15^N values of muscle tissue and gut contents of the additional *N. melanostomus* samples followed similar functions and were strongly dependent on *L*_T_ (Figure 4). Both datasets from the established area (muscle tissue: R^2^ = 0.541, p<0.001; gut content: R^2^ = 0.306, p<0.001) and the IF2010 (muscle tissue: R^2^ = 0.213, p<0.001; gut content: R^2^ = 0.161, p<0.001) were highly significantly described by parabolic regressions with size and diet-tissue shifts of 3.1 ‰ (SE 0.3 ‰) in case of the established area and 4.7 ‰ (SE 0.2 ‰) in case of the IF2010. These mean diet-tissue shifts were significantly (Mann-Whitney U; p<0.001) different and the residuals of the regressions indicated that diet and muscle were predicted equally well with a slight parabolic trend in the residuals. The δ^15^N value of the gut content of *N. melanostomus* changed with *L*_T_ during the observed growth-phases. In the established population, δ^15^N values increased by about 2.5 ‰ up to a *L*_T_ of 10 cm, and then decreased again, while δ^15^N values increased by about 0.8 ‰ up to a *L*_T_ of 12.5 cm, and then slightly decreased in the IF2010. Notably, the mean δ^15^N value of the gut contents was calculated from the mean δ^15^N values of the detected species and thus reflects the change in the composition of the prey species but not an isotopic change within the individual prey species.

The mean δ^13^C values did not significantly differ between the investigated populations in both sexes; however a significant (Mann-Whitney U; p<0.01) sex-specific difference was observed in the IF2009.

The mean HSI significantly differed between the investigated populations both in females (Kruskal-Wallis; p<0.001) and males (Kruskal-Wallis; p<0.01), with highest values at the IF2010. Compared with the IF2009, mean HSI in females was significantly higher in the IF2010 (Mann-Whitney U; p<0.001) and the established area (Mann-Whitney U; p<0.05). In females, the mean HSI was significantly (Mann-Whitney U; p<0.001) higher in the IF2010 than in the established area. In males, the mean HSI was significantly higher at the IF2010 (Mann-Whitney U; p<0.05) and the established area (Mann-Whitney U; p<0.01) compared with the IF2009, whereas no significant difference was observed between the IF2010 and the established area. In females, the mean HSI was significantly (Mann-Whitney U; p<0.05) higher (about 20%) compared to males in the IF2010.

The *I*_SF_ did not significantly differ between the investigated populations. No significant sex-specific differences were observed within the mean *I*_SF_ within the populations, indicating a similar feeding status.

### Benthic invertebrate availability

The benthic invertebrate community mainly consisted of highly abundant amphipods (*Dikerogammarus spp.*, *Chelicorophium spp.*, *Iaera spp.),* molluscs (*Dreissena spp.*, *Corbicula spp., Potamopyrgus spp.*) and other exotic species, primarily originating from the Ponto-Caspian area. Overall, alien species comprised more than 50% of all taxa and about 90% of *I*_FI_. However no differences in *I*_FI_ could be observed between the analyzed areas. In contrast to molluscs and amphipods, all Ephemeroptera, Trichoptera and Plecoptera (EPT) were indigenous and part of the typical and original fauna. The mean *I*_FI_ of EPT significantly (Kruskal-Wallis; p<0.01) differed between the investigated areas. The mean *I*_FI (EPT)_ was significantly (Mann-Whitney U; p<0.01) higher in the IF2010 (about 13-fold) and the IF2009 (about 9-fold, but not significantly) compared with the established area. Except for the cumulative category EPT, no significant differences in both CPUE and *I*_EI_ of all other benthic invertebrate taxa were observed between the analyzed river stretches. The mean CPUE_(EPT)_ significantly (Kruskal-Wallis; p<0.001) differed between the investigated areas. At the IF2010, the mean CPUE was both significantly (Mann-Whitney U; p<0.001) higher than in the IF2009 (about 5-fold) and the established area (about 8-fold). No significant difference in the mean CPUE_(EPT)_ was observed between the IF2009 and the established area. The mean *I*_EI (EPT)_ significantly (Kruskal-Wallis; p<0.001) differed between the investigated areas, with highest values at the IF2010 and lowest values in the established area. The mean *I*_EI (EPT)_ of the IF2010 was significantly (Mann-Whitney U; p<0.001) higher (30-fold) compared with the established area and the IF2009 (3-fold), but not significantly. The mean *I*_EI (EPT)_ did not significantly differ between IF2009 and the established area.

### Parasitic load

Indicators for endoparasitic acanthocephala infection (i.e. abundance and density) significantly (Kruskal-Wallis; p<0.001) differed between the investigated areas in females and males, with highest values at the IF2010. At the IF2010, the mean acanthocephalan abundance in females was both significantly (Mann-Whitney U; p<0.001) higher compared with in the IF2009 (about 5-fold) and the established area (about 2-fold). The mean abundance of acanthocephalans in males from the IF2010 was significantly (Mann-Whitney U; p<0.001) higher (about 6-fold) compared to the IF2009 and significantly (Mann-Whitney U; p<0.01) higher compared to the established area (about 2-fold). Males from the IF2009 had a significantly (Mann-Whitney U; p<0.01) lower (3-fold) mean abundance of acanthocephalans than males from the established population. The only sex-specific difference was observed in the IF2010, where females had a significantly (Mann-Whitney U; p<0.01) higher mean abundance of acanthocephalans than their male conspecifics.

At the IF2010, the mean density of acanthocephalans in females was both significantly (Mann-Whitney U; p<0.001) higher compared with in the IF2009 (about 5-fold) and the established area (about 2-fold). Also in males, the mean density of acanthocephalans from the IF2010 was significantly (Mann-Whitney U; p<0.001) higher (about 6-fold) compared with the IF2009 and significantly (Mann-Whitney U; p<0.01) higher compared to the established area (about 2-fold).

Males from the IF2009 had a significantly (Mann-Whitney U; p<0.01) lower (3-fold) mean density of acanthocephalans than males from the established area. Females had significantly (Mann-Whitney U; p<0.05) higher densities of acanthocephalans than males in both the IF2009 (2-fold) and the established area (1.4-fold). The mean abundance of ectoparasites of the genus *Rossicotrema spp.* (Plathyhelminthes) was generally low and did neither significantly differ between specimens of the populations analyzed nor between sexes (Table 4).

## Discussion

This study provides evidence of differences in demography and sex ratio, morphology, feeding behaviour and parasitic load of invasive round gobies among specimens sampled at an invasion front and those from the established area. These results support the previously suggested plasticity of this species based on comparisons of population data from the native range with those from invaded areas [8], [16], [19]. Pioneering populations from the invasion front were female-dominated, comprising large sized, heavier individuals with highest condition and lowest gonado-somatic index. At the established area, *N. melanostomus* revealed an ontogenetic diet shift with a switch from preying upon insects and crustaceans to a mainly mollusc dominated diet at a *L*_T_ of 10.0 cm. In contrast, the pioneering population (IF2010) exhibited a less pronounced, more continuous diet shift with a deferred and weaker diet switch at a larger size of about 12.5 cm. According to the “enemy-release-hypothesis”, lower abundance and density of endoparasites would have been expected at the invasion front. Instead, opposite results of higher acanthocephalan loads were observed in both sexes at the invasion front (IF2010) and seemingly did not hamper invasion success. Compared with the established area, CPUE and *I*_FI_ of indigenous Ephemeroptera, Trichoptera and Plecoptera was higher at the invasion front, whereas no differences in both CPUE and *I*_EI_ of all other benthic invertebrate taxa were observed between all analyzed river stretches. Generally, the IF2009 behaved intermediately, with characteristics of both the IF2010 and the established population, underlining the high pace of the observed invasion processes. Overall, the pronounced changes in fish and invertebrate communities with a dominance of alien species suggest an invasional meltdown and a shift of the upper Danube River towards a novel ecosystem with species that have greater resistance to goby predation. This seems to contribute to overcoming biological resistance and improve rapidity of dispersal.

### Increased competitive ability

In line with our initial hypothesis, female and male round gobies from the invasion front were bigger (larger and heavier), revealing higher condition factors than those from established areas. These characteristics probably increase their performance and competitive ability, also reducing predation risk at the early stages of the invasion process. In turn, this can contribute to better chances for establishment and further spread. The greater availability of prey and a smaller degree of intraspecific competition in novel areas of distribution may also contribute to this pattern. Generally, females had a higher condition than males, but did not differ in body-size and weight. In line with our findings, Gutowsky and Fox (2011) [8] also caught the largest individuals of each sex at initially invaded areas, but found significantly larger males than females at the edges of upstream expansion areas. This size-specific difference may result from using angling as a sampling method by these authors since angling was found to be selective for larger males [65]. In contrast to our findings, Brownscombe and Fox (2012) [16] caught smaller round gobies at recently invaded areas compared to longer established sites, indicating that local habitat conditions and community structure can strongly influence trait selection.

According to the results of our study, five to seven years after introduction, males from the established area seem to grow larger and become heavier than females, reflecting observed sexual dimorphism and indicating major changes within invasive populations of round goby over time. In contrast to the increased somatic growth in initially invaded areas, the lower GSI of females at the invasion front compared to established areas suggests that somatic performance seems to be more important than investment in reproduction during the early stages of the invasion process. It needs to be noted, however, that egg size may also play a role since individuals with the same GSI but different egg sizes can produce different numbers of offspring. Such relationships between fecundity, egg size and juvenile performance are well known in many fish species [66], however, there is no clear association between egg size and maximum body length of newborn gobiids [67]. Males had a similar reproductive power along the invasion pathway (constant by time), while fecundity of females increased over time since invasion. A similar pattern had been reported from the Trent-Severn-Waterway, where GSI in female round gobies increased by time, too [19]. A possible explanation may be that the age structure of the studied populations changed over time, with increased fecundity in larger and older females which occur at higher frequency later in the year.

In case of plants, an “*evolution of increased competitive ability”* has been proposed, suggesting that specimens produce more seeds or grow more vigorous and taller in environments outside their native ranges [52], [68]. This concept also seems applicable to round goby and may explain the invasive success of this species, particularly in the early stages of the invasion process. In the case of gobies, stronger emphasis seems to be put on growth-related traits instead of reproductive traits to increase competitive ability. Also, the fact that they are dispersing into a highly altered environment containing alien but familiar food resources could play a role.

### Effects on the food web

Different feeding strategies of invasive round goby were detected between established and pioneering populations. According to the stable isotope analyses, only females and males from the IF2010 utilized similar food resources, as evident from both similar δ^15^N and δ^13^C signatures indicating the same trophic niche. A clear sex-specific difference was observed both in the established population, indicated by different δ^15^N values with no difference in δ^13^C values, and in the IF2009, indicated by different δ^13^C values with no difference in δ^15^N values. Such sex-specific signatures could result from selective feeding or competition between males and females under food-resource limitation (the latter seems unlikely because no differences in the distribution and abundance of benthic invertebrates had been found except for EPT). These sex-specific differences in SIA could also derive from different habitat utilisation, thus indicating beginning habitat saturation.

The δ^15^N signatures of the recently invaded IF2010 and IF2009 exceeded the values of the established area indicating a slightly higher trophic level there, which may result from a targeting of more valuable larger-sized, energetically enriched prey.

Food web baseline variation between the river stretches (i.e. the mean decrease/increase in δ^15^N values of the primary consumers *D. polymorpha* and *C. fluminalis* [69]), which was not corrected for, only played a minor role, as δ^15^N values (mean = 9.91, SD = 0.31) had a very narrow range.

At the established area, *N. melanostomus* exhibits a pronounced and continuous ontogenetic diet shift, which determines a broad dietary niche at the population level. At a total length of about 10 cm, it switches from preying upon insects and crustaceans (increasing limb, Figure 4) to a mainly mollusc dominated diet (decreasing limb, Figure 4), which can also be interpreted as an increasing specialization at the individual level [13]. The IF2010 population exhibits a less pronounced, more continuous diet shift, indicating a narrower dietary niche. At a total length of about 12.5 cm, *N. melanostomus* tend to switch from an amphipod-based diet (increasing limb, Figure 4) to some preying upon molluscs (decreasing limb, Figure 4), which mirrors a high preference towards amphipods under conditions of low intraspecific competition. This plasticity within an ontogenetic determined behaviour may contribute to the high invasion success.

The decreasing trend in CPUE and *I*_EI_ in EPT by time, with highest values at the IF2010 clearly highlights the impact of round goby on native biodiversity, similar to the one described by Kipp and Ricciardi (2012) [25] for North American rivers. Underlining the extraordinary preference of round goby for these native taxa, the *I*_FI (EPT)_ by far exceeded the *I*_EI (EPT)_ in all round goby populations (Table 4).

Along the 200 river-km invasion pathway of round goby within the upper Danube River, a benthic-invertebrate community, highly dominated by non-native species (similar in abundance and distribution) had already been established before round goby invaded. This highly altered benthic invertebrate community with an aquatic fauna typical for lower sections of streams, found for the whole investigated stretch of the upper Danube River, corroborates analogous findings from the River Rhine [69]. Round goby and other Ponto-Caspian neogobiids rather seem to complete a faunistic homogenization of such large rivers [70] than they would represent an independent single species invasion phenomenon. The observed impact on EPT may underline this ongoing process, described as an “invasional meltdown” [71], which already seemed to happen in an advanced stage at the upper Danube River. Such an invasional meltdown scenario was also discussed for the Laurentian Great Lakes area [72], indicating similar conditions and developments there.

### Sex ratio

Although males revealed a more exploratory behavior and greater moving distances in recent studies [73], round goby populations at invasion fronts appear to be female-biased ([16], [18] this study), while established populations seem to be typically male-dominated (Trent River [8], [31]; Lake Ontario [74]; Gulf of Gdansk, Baltic Sea [21]). This observation is in line with this study, as four out of five first recorded invaders in autumn 2010 (right shoreline) and seven out of thirteen pioneers in autumn 2011 (right shoreline) were female at the IF2010, suggesting that a higher proportion of females may contribute to range expansion in round goby. Despite the sex-selectivity of the different sampling techniques used, which is higher in hook-and-line based sampling than in electrofishing as applied in this study (see Brandner et al., 2013 [65]), migrating adult females appear to be a main driver of range expansion. Among various reasons, inbreeding depression avoidance, asymmetry in the costs of dispersal and mating system characteristics [75] can cause sex-biased dispersal in invasions. Since male round gobies invest more energy in parental care and territorial defense than females, sex-biased dispersal by females could also be a possible strategy to first, reduce intraspecific competition for mates among females and second, to benefit from a lower predation risk at the invasion front [76], which might be especially true for larger individuals.

### Parasitic load

Fish parasites of the genus Acanthocephala, which are specific endoparasites with a complex life-cycle, were surprisingly found in highest densities and abundance in goby-specimens from IF2010, while unspecific ectoparasites (*Rossicotrema spp.*) were equally distributed in very low abundance and densities among gobies along the whole invasion pathway. Finding highest abundance and density of Acanthocephala in round goby from the invasion front compared to the other investigated areas contradicts our hypothesis and the “enemy release hypothesis”. In the complex life-cycle of acanthocephalans, amphipods serve as highly species-specific intermediate hosts [77]. A high proportion of amphipods in the diets can lead to high infection rates of Acanthocephala in *N. melanostomus*, but simultaneously also to high values of lipid storage due to the nutritive value of the consumed prey. Thus even heavy acanthocephalan infections are unlikely to have a large pathogenic effect in gobies [58]. Consequently, the highest values of HSI, observed in the specimens from the IF2010 and IF2009 can probably be explained by better feeding conditions in these areas. Both, the higher HSI as a short-term indicator and the higher abundance of Acanthocephala as a long-term indicator mirror a better energetic status and probably higher fitness displayed by higher *K* in gobies from the invasion front compared with established populations. This seems especially to be true for females compared to their male conspecifics (Table 4). Since no difference had been observed in the degree of stomach fullness between all populations, no symptom for food limitation was observed using this metric.

Acanthocephala are also known to possess the ability to induce behavioural changes in their intermediate hosts, which increase the likelihood of becoming a prey for a fish [78]. Since abundance of amphipods was equally distributed among the areas investigated, at the invasion front a smaller number of gobies can therefore choose among a relatively higher number of amphipods, possibly effectively selecting for infected intermediate hosts. In case of equally distributed infected intermediate hosts, a small number of gobies will acquire a higher number of acanthocephalans in areas with low goby abundance. Consequently, this effect also indicates an unlimited, ‘free-to-choose’ availability of high valuable food- resources at an invasion front.

### Time trends

The invasion in the upper Danube River can be considered a fast process. During study initiation, the most upstream located sampling stretch (#10 “Kelheim”) was intended to serve as a negative control area, free of round goby. However, *N. melanostomus* was established at the invasion front of the year 2009 (#09 “Bad Abbach”) within two years, and successfully invaded the projected negative control by upstream migration within one year. Due to their benthic morphology and their small home range, round gobies would be expected to have a poor natural dispersal ability, especially in upstream direction [79], [80]. This study corroborates recently reported fast spread-rates with estimates ranging from 500 m year^−1^on average [30] to up to 1–4 km year^−1^ in selected areas [26]. Since this study indicated a spread rate, being up to four-times higher by covering even a distance of about 17 river-km in about one year, the high pace of round goby invasion might have been underestimated. Similarly, Brownscombe et al. (2012) [31] calculated dispersal rates of 5 to 27 km year^−1^ using gamma distribution models. Generally, round goby riverine colonization appears to be driven by ‘stratified dispersal’, a strategy combined of contiguous diffusion over short distances by most individuals and long-distance colonization (jump events) by migrant individuals [26], [30]. Given a low sampling bias [65] and a minimum population doubling time (estimates based on empirical models) of 1.4 to 4.4 years [81], the danubian invasion of *N. melanostomus* seems to be mainly driven by a high upstream directed propagule pressure from densely populated established areas with strong, large sized individuals, rather than by an increased reproductive success at the invasion front. The anti-cyclical trend between round goby density increase and population decrease of the two most abundant autochthonous fish species (barbel and chub) and EPT observed in this study (Tables 2 and 4), suggests alterations of the food web. It needs to be noted however, that the causality of these relationships needs further testing since no evidence for preying on eggs and larvae of other fishes was detectable.

The increasing trend in the CPUE at the established area (Table 2) indicates that the population density is still increasing there (73% to the total catch in the analyzed rip-rap mesohabitat), suggesting that the carrying capacity has not being reached yet. Round goby populations in Hamilton Harbour (North-American Great Lakes area) reached saturation densities approximately one decade after arrival, with densities being about 50% greater than the expected carrying capacity [82].

## Conclusions

In this study, an upstream-directed colonization of *N. melanostomus* along a fluvial gradient with a distinct invasion front was observed, from total absence until establishment. Competitive ability and invasion success of the gobies at the invasion front seems to be largely determined by somatic investment (“bigger is better”) instead of reproductive investment. The larger size and higher condition factor of gobies at the invasion front compared to those at established areas can be explained by less limited food resources in newly invaded areas. The finding of higher parasitic load at the invasion front was surprising and in contrast to expectations according to the “enemy release hypothesis”, indicating that this factor is less important. The resulting pronounced changes in fish and invertebrate communities induced by the goby invasion suggest the occurrence of an invasional meltdown and a shift of the upper Danube River towards a novel ecosystem with communities and species that have greater resistance to goby predation. This seems to contribute to overcoming biological resistance and improve rapidity of dispersal. Such a complex change is also along the lines of what is happening to other aquatic systems in the world, i.e. the creation of novel ecosystems through the combination of environmental change and the impact of invasive species [83], [84]. As a result, novel ecosystems may provide different functional properties and ecosystem services, even though their persistence and values remain largely unknown [84]. This also appears to be true for the Danube River, where we observed a rapid ongoing shift from indigenous biodiversity towards a ubiquitous faunistic complex of potentially co-evolved exotic species which are adapted to human-altered aquatic systems. Consequently, especially the success of Ponto-Caspian invaders reflects fundamental ecological changes in the large European freshwater ecosystems [13], which make a return to original communities almost impossible. This also questions the use of historical reference conditions and communities as a conservation target, e.g. in the context of the European Water Framework Directive and the development of any other conservation target.
